# Crystal structure of bromido-*fac*-tricarbon­yl[5-phenyl-3-(pyridin-2-yl)-1*H*-1,2,4-triazole-κ^2^ *N*,*N*′]rhenium(I)

**DOI:** 10.1107/S1600536814025604

**Published:** 2014-11-29

**Authors:** Kseniia Piletska, Konstantin V. Domasevitch, Alexander V. Shtemenko

**Affiliations:** a Department of Inorganic Chemistry, Ukrainian State University of Chemical Technology, Gagarin Ave. 8, Dnipropetrovsk 49005, Ukraine; b Inorganic Chemistry Department, National Taras Shevchenko University of Kyiv, Volodymyrska Street 64/13, Kyiv 01601, Ukraine

**Keywords:** Crystal structure, rhenium carbonyl complex, 5-phenyl-3-(pyridin-2-yl)-1*H*-1,2,4-triazole, hydrogen bonding, slipped π–π stacking inter­actions

## Abstract

In the title compound, [ReBr(C_13_H_10_N_4_)(CO)_3_], the Re^I^ atom has a distorted octa­hedral coordination environment. Two N atoms of the 5-phenyl-3-(pyridin-2-yl)-1*H*-1,2,4-triazole ligand and two of the three carbonyl groups occupy the equatorial plane of the complex, with the third carbonyl ligand and the bromide ligand in the axial positions. The three carbonyl ligands are arranged in a *fac* configuration around the Re^I^ atom. Mutual N—H⋯Br hydrogen bonds arrange mol­ecules into centrosymmetric dimers. Additional stabilization within the crystal structure is provided by C—H⋯O and C—H⋯Br hydrogen bonds, as well as by slipped π–π stacking inter­actions [centroid-to-centroid distance = 3.785 (5) Å], defining a three-dimensional network.

## Abstract 2

In this rhenium carbonyl complex of 5-phenyl-3-(pyridin-2-yl)-1*H*-1,2,4-triazole, the Re^I^ atom has a distorted octa­hedral coordination environment. Mutual N—H⋯Br hydrogen bonds arrange the mol­ecules into centrosymmetric dimers. Additional stabilization within the crystal structure is provided by C—H⋯O and C—H⋯Br hydrogen bonds, as well as by slipped π–π stacking inter­actions.

## Chemical context   

The coordination chemistry of rhenium and technetium has been well studied over the last half century, particularly in view of the potential applications of their ^186/188^Re and ^99m^Tc isotopes in therapeutic and diagnostic agents in nuclear medicine (Volkert & Hoffman, 1999 ▶; Alberto *et al.*, 1999 ▶). Complexes of the type [*M*(CO)_3_(*NN*)*X*] (*M* = Tc, Re; *NN* = bidentate nitro­gen donor; *X* = anionic ligand) have been shown to possess inter­esting photophysical, photochemical and excited-state redox properties (Striplin & Crosby, 2001 ▶; Stufkens & Vlcěk, 1998 ▶), making this class of complexes applicable as fluorescent probes, in addition to their potential usage as radio-imaging and therapeutic agents. Moreover, metal carbonyls display intense infrared absorptions in the range 1800 to 2200 cm^−1^, which is the IR transparency window for biological media (Hildebrandt, 2010 ▶). In addition to their luminescent properties, the vibrational signature of *fac*-[Re(CO)_3_(*NN*)] is appropriate for IR imaging (Policar *et al.*, 2011 ▶; Clède *et al.*, 2012 ▶). They are thus valuable as small mol­ecular units enabling multimodal imaging involving vibrational-based detections (IR, Raman) and fluorescence (Clède *et al.*, 2012 ▶). In [Re(CO)_3_(*NN*)*X*] compounds, the photophysical properties of the complexes are closely dependent on the ligand. When *NN* is a ligand with low π* orbitals, the corresponding [Re(CO)_3_(*NN*)] unit is luminescent (Wrighton & Morse, 1974 ▶) and this property has often been used in subcellular bio-imaging (Lo *et al.*, 2012 ▶; Baggaley *et al.*, 2012 ▶; Xiang *et al.*, 2013 ▶; Coogan & Fernandez-Moreira, 2014 ▶).

In this communication, we report the synthesis and crystal structure analysis of a novel Re^I^ complex which contains the triazole ligand 5-phenyl-3-(pyridin-2-yl)-1*H*-1,2,4-triazole, [Re(CO)_3_(C_13_H_10_N_4_)Br]. Its luminescent properties will be reported in a forthcoming article.
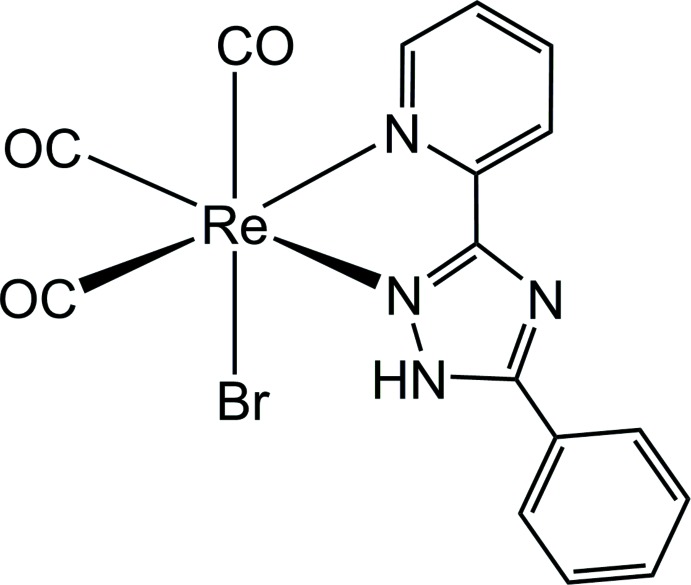


## Structural commentary   

**Figure 1:**
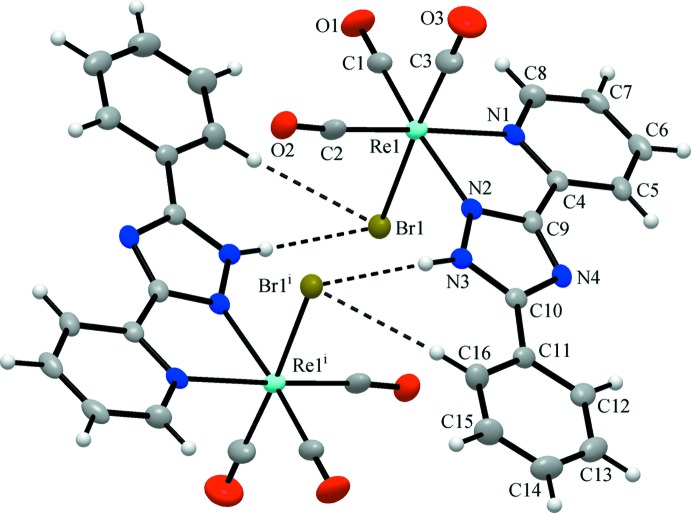
The structure of the title complex, showing the association of mol­ecules into a centrosymmetric dimer by means of mutual hydrogen bonds of the N—H⋯Br and C—H⋯Br types. Displacement ellipsoids are drawn at the 40% probability level. [Symmetry code: (i) −*x* + 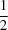, −*y* + 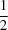, −*z*.]

In the title compound, the Re^I^ atom is in a slightly distorted octa­hedral coordination environment (Fig. 1 ▶). The three carbonyl ligands bonded to the Re^I^ atom are arranged in a *fac*-configuration. The distances of C1, C2, and C3 to the Re^I^ atom are 1.905 (4), 1.915 (4), and 1.922 (6) Å, respectively, and the Re—N bonds lengths are 2.201 (3) and 2.164 (3) Å. The CO ligands are almost linearly coordinated with O—C—Re bond angles of 178.4 (4), 175.6 (3) and 179.0 (4)°. The C—Re—C bond angles between CO carbon atoms are 87.78 (17), 90.4 (2) and 89.18 (19)°, close to ideal values, whereas the *cis* equatorial bite angle [N1—Re1—N2] is 74.33 (11)°. All other bond lengths and angles are comparable to those found for related Re^I^ complexes (Rajendran *et al.*, 2000 ▶).

## Supra­molecular features   

**Figure 2:**
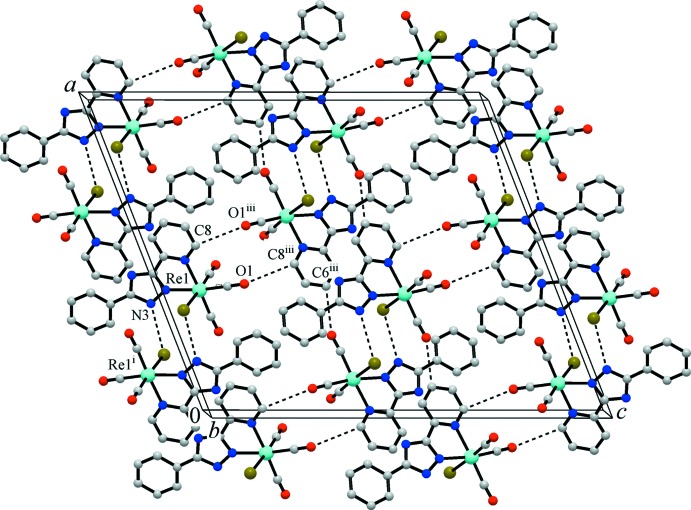
The crystal structure of the title complex, showing weak hydrogen-bonding inter­actions (indicated by dotted lines) of the type C—H⋯O between carbonyl O atoms and pyridyl C—H groups of the organic ligands. [Symmetry codes: (i) −*x* + 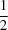, −*y* + 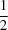, −*z*; (iii) −*x* + 1, *y*, −*z* + 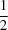.]

The title compound adopts a typical mol­ecular structure. There is only one relatively strong donor (N—H) and one acceptor (Br) site for hydrogen-bonding inter­actions, which arrange mol­ecules into dimers (Table 1 ▶, Fig. 1 ▶). Weak hydrogen bonds of the type C—H⋯O with carbonyl O atoms as acceptor groups play a supporting role in the crystal packing. Nevertheless, these inter­actions demonstrate a clear discrimination of the C—H binding sites that follow a common pattern. The C—H⋯O hydrogen bonds present are provided by the 2- and 4-C—H protons of the pyridine ring, which are the most polarized and acidic. Besides C—H⋯Br inter­actions, weak slipped π–π stacking inter­actions between pyridine and phenyl rings (symmetry code: 1 − *x*, −*y*, −*z*) [with a shortest separation of C6⋯C11(1 − *x*, −*y*, −*z*) = 3.265 (6) Å, a centroid-to-centroid distance of 3.785 (5) Å and an inter­planar angle of 7.1 (3)°] also appear to be involved in the stabilization of the crystal structure (Fig. 2 ▶).

## Synthesis and crystallization   

Penta­carbonyl­rhenium(I) bromide (0.1 g, 0.246 mmol) was reacted with 5-phenyl-3-(pyridin-2-yl)-1*H*-1,2,4-triazole (0.1 g, 0.492 mmol) in benzene at 353 K, with stirring, under a steady stream of argon for five h. The dark-yellow solution was removed from the heat and allowed to cool overnight. The yellow product was collected by suction filtration, washed with a 50 ml portion of petroleum ether and dried. Yield = 0.107g, (76.4%). Crystals suitable for X-ray diffraction were obtained by slow diffusion of hexane into a methanol solution of the complex. IR (KBr, cm^−1^): ν_as_(CO) 2028 (*s*), ν_s_(CO) 1912 (*s*).

## Refinement   

Crystal data, data collection and structure refinement details are summarized in Table 2 ▶. H atoms were positioned with idealized geometry and were refined with C—H = 0.94, N—H = 0.87 Å and *U*_iso_(H) = 1.2*U*_eq_(C,N).

## Supplementary Material

Crystal structure: contains datablock(s) I. DOI: 10.1107/S1600536814025604/wm5089sup1.cif

Structure factors: contains datablock(s) I. DOI: 10.1107/S1600536814025604/wm5089Isup2.hkl

CCDC reference: 1031232

Additional supporting information:  crystallographic information; 3D view; checkCIF report

## Figures and Tables

**Table 1:** Hydrogen-bond geometry (, )

*D*H*A*	*D*H	H*A*	*D* *A*	*D*H*A*
N3H3Br1^i^	0.87	2.51	3.360(3)	168
C16H16Br1^i^	0.94	2.87	3.784(4)	165
C6H6O2^ii^	0.94	2.38	3.194(5)	145
C8H8O1^iii^	0.94	2.56	3.285(5)	134

Symmetry codes: (i) 
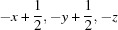
; (ii) 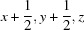; (iii) 
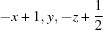
.

**Table 2:** Experimental details

Crystal data	
Chemical formula	[ReBr(C_13_H_10_N_4_)(CO)_3_]
*M*_r_	572.39
Crystal system, space group	Monoclinic, *C*2/*c*
Temperature (K)	213
*a*, *b*, *c* ()	20.8082(15), 7.2521(4), 24.386(2)
()	111.599(7)
*V* (^3^)	3421.5(4)
*Z*	8
Radiation type	Mo *K*
(mm^1^)	9.46
Crystal size (mm)	0.14 0.12 0.11

Data collection	
Diffractometer	Stoe Imaging plate diffraction system
Absorption correction	Numerical (*X-RED* and *X-SHAPE*; Stoe Cie, 2001 ▶)
*T*_min_, *T*_max_	0.319, 0.385
No. of measured, independent and observed [*I* > 2(*I*)] reflections	14578, 4092, 2844
*R*_int_	0.057
(sin /)_max_ (^1^)	0.661

Refinement	
*R*[*F* ^2^ > 2(*F* ^2^)], *wR*(*F* ^2^), *S*	0.023, 0.050, 0.84
No. of reflections	4092
No. of parameters	226
H-atom treatment	H-atom parameters constrained
_max_, _min_ (e ^3^)	1.03, 1.14

Computer programs: *IPDS* (Stoe Cie, 2001 ▶), *SHELXS97* and *SHELXL2014* (Sheldrick, 2008 ▶), *DIAMOND* (Brandenburg, 1999 ▶) and *WinGX* (Farrugia, 2012 ▶).

## supplementary crystallographic information

### Crystal data

**Table:**

[ReBr(C_13_H_10_N_4_)(CO)_3_]	*F*(000) = 2144
*M**_r_* = 572.39	*D*_x_ = 2.222 Mg m^−^^3^
Monoclinic, *C*2/*c*	Mo *K*α radiation, λ = 0.71073 Å
*a* = 20.8082 (15) Å	Cell parameters from 8000 reflections
*b* = 7.2521 (4) Å	θ = 3.0–28.0°
*c* = 24.386 (2) Å	µ = 9.46 mm^−^^1^
β = 111.599 (7)°	*T* = 213 K
*V* = 3421.5 (4) Å^3^	Prism, yellow
*Z* = 8	0.14 × 0.12 × 0.11 mm

### Data collection

**Table:**

Stoe Imaging plate diffraction system diffractometer	2844 reflections with *I* > 2σ(*I*)
Radiation source: fine-focus sealed tube	*R*_int_ = 0.057
φ oscillation scans	θ_max_ = 28.0°, θ_min_ = 3.0°
Absorption correction: numerical (*X-RED* and *X-SHAPE*; Stoe & Cie, 2001)	*h* = −27→27
*T*_min_ = 0.319, *T*_max_ = 0.385	*k* = −9→8
14578 measured reflections	*l* = −32→32
4092 independent reflections	

### Refinement

**Table:**

Refinement on *F*^2^	Primary atom site location: structure-invariant direct methods
Least-squares matrix: full	Secondary atom site location: difference Fourier map
*R*[*F*^2^ > 2σ(*F*^2^)] = 0.023	Hydrogen site location: inferred from neighbouring sites
*wR*(*F*^2^) = 0.050	H-atom parameters constrained
*S* = 0.84	*w* = 1/[σ^2^(*F*_o_^2^) + (0.0225*P*)^2^] where *P* = (*F*_o_^2^ + 2*F*_c_^2^)/3
4092 reflections	(Δ/σ)_max_ = 0.001
226 parameters	Δρ_max_ = 1.03 e Å^−^^3^
0 restraints	Δρ_min_ = −1.14 e Å^−^^3^

### Special details

**Table:**

Geometry. All e.s.d.'s (except the e.s.d. in the dihedral angle between two l.s. planes) are estimated using the full covariance matrix. The cell e.s.d.'s are taken into account individually in the estimation of e.s.d.'s in distances, angles and torsion angles; correlations between e.s.d.'s in cell parameters are only used when they are defined by crystal symmetry. An approximate (isotropic) treatment of cell e.s.d.'s is used for estimating e.s.d.'s involving l.s. planes.

### Fractional atomic coordinates and isotropic or equivalent isotropic displacement parameters (Å^2^)

**Table:**

	*x*	*y*	*z*	*U*_iso_*/*U*_eq_	
Re1	0.38382 (2)	0.25265 (3)	0.10559 (2)	0.02798 (5)	
Br1	0.32731 (2)	0.54427 (7)	0.04146 (2)	0.03557 (11)	
O1	0.40827 (17)	0.4654 (6)	0.21974 (13)	0.0594 (10)	
O2	0.24980 (15)	0.1146 (5)	0.11477 (13)	0.0516 (10)	
O3	0.4540 (2)	−0.0838 (7)	0.17525 (18)	0.0796 (14)	
N1	0.48045 (14)	0.3439 (5)	0.09681 (13)	0.0264 (7)	
N2	0.38108 (14)	0.1420 (5)	0.02237 (12)	0.0244 (7)	
N3	0.33622 (14)	0.0546 (5)	−0.02600 (12)	0.0258 (7)	
H3	0.2960	0.0108	−0.0295	0.031*	
N4	0.42685 (15)	0.1255 (5)	−0.04749 (13)	0.0255 (7)	
C1	0.3988 (2)	0.3830 (7)	0.17704 (17)	0.0372 (12)	
C2	0.2983 (2)	0.1674 (7)	0.10904 (15)	0.0361 (11)	
C3	0.4289 (2)	0.0391 (9)	0.14970 (19)	0.0463 (13)	
C4	0.49040 (18)	0.2879 (5)	0.04763 (16)	0.0246 (9)	
C5	0.54965 (18)	0.3314 (7)	0.03688 (17)	0.0297 (9)	
H5	0.5554	0.2910	0.0024	0.036*	
C6	0.59958 (19)	0.4352 (7)	0.07811 (18)	0.0341 (10)	
H6	0.6407	0.4653	0.0725	0.041*	
C7	0.58898 (19)	0.4942 (6)	0.12745 (18)	0.0347 (10)	
H7	0.6225	0.5668	0.1557	0.042*	
C8	0.5289 (2)	0.4467 (7)	0.13551 (17)	0.0340 (10)	
H8	0.5220	0.4883	0.1694	0.041*	
C9	0.43441 (18)	0.1817 (6)	0.00714 (16)	0.0258 (8)	
C10	0.36475 (18)	0.0474 (6)	−0.06765 (15)	0.0246 (8)	
C11	0.33235 (18)	−0.0329 (6)	−0.12595 (15)	0.0268 (9)	
C12	0.3677 (2)	−0.0228 (7)	−0.16469 (17)	0.0337 (10)	
H12	0.4104	0.0389	−0.1534	0.040*	
C13	0.3397 (3)	−0.1037 (7)	−0.21942 (18)	0.0426 (12)	
H13	0.3636	−0.0967	−0.2454	0.051*	
C14	0.2775 (3)	−0.1946 (7)	−0.23681 (19)	0.0448 (12)	
H14	0.2592	−0.2506	−0.2741	0.054*	
C15	0.2425 (2)	−0.2025 (7)	−0.19894 (19)	0.0403 (12)	
H15	0.1998	−0.2636	−0.2106	0.048*	
C16	0.2693 (2)	−0.1219 (7)	−0.14401 (18)	0.0369 (11)	
H16	0.2446	−0.1276	−0.1187	0.044*	

### Atomic displacement parameters (Å^2^)

**Table:**

	*U*^11^	*U*^22^	*U*^33^	*U*^12^	*U*^13^	*U*^23^
Re1	0.02700 (7)	0.03224 (11)	0.02386 (7)	−0.00874 (8)	0.00836 (5)	0.00069 (8)
Br1	0.03230 (18)	0.0375 (3)	0.03867 (19)	−0.0024 (2)	0.01516 (15)	0.0035 (2)
O1	0.068 (2)	0.075 (3)	0.0338 (16)	−0.021 (2)	0.0179 (15)	−0.0196 (19)
O2	0.0364 (15)	0.077 (3)	0.0440 (17)	−0.0237 (18)	0.0178 (13)	−0.0038 (18)
O3	0.081 (3)	0.071 (4)	0.079 (3)	0.009 (3)	0.020 (2)	0.034 (3)
N1	0.0216 (14)	0.022 (2)	0.0309 (15)	−0.0032 (15)	0.0036 (12)	0.0023 (15)
N2	0.0224 (14)	0.019 (2)	0.0291 (15)	−0.0061 (14)	0.0071 (12)	0.0002 (14)
N3	0.0250 (14)	0.023 (2)	0.0270 (14)	−0.0053 (15)	0.0074 (11)	−0.0012 (15)
N4	0.0240 (14)	0.018 (2)	0.0350 (16)	−0.0011 (14)	0.0116 (12)	0.0018 (15)
C1	0.032 (2)	0.049 (4)	0.0275 (19)	−0.009 (2)	0.0074 (15)	0.004 (2)
C2	0.036 (2)	0.047 (3)	0.0221 (17)	−0.013 (2)	0.0068 (15)	−0.0053 (19)
C3	0.041 (2)	0.053 (4)	0.043 (2)	−0.007 (3)	0.0144 (19)	0.017 (3)
C4	0.0250 (16)	0.015 (3)	0.0322 (17)	0.0002 (15)	0.0084 (13)	0.0071 (15)
C5	0.0245 (17)	0.023 (3)	0.042 (2)	0.0009 (18)	0.0128 (16)	0.009 (2)
C6	0.0243 (18)	0.021 (3)	0.052 (2)	−0.0034 (19)	0.0086 (17)	0.014 (2)
C7	0.0268 (18)	0.018 (3)	0.047 (2)	−0.0065 (18)	−0.0005 (16)	0.009 (2)
C8	0.038 (2)	0.024 (3)	0.0353 (19)	−0.007 (2)	0.0078 (16)	−0.003 (2)
C9	0.0266 (17)	0.016 (2)	0.0354 (19)	0.0016 (17)	0.0123 (15)	0.0062 (17)
C10	0.0298 (18)	0.013 (2)	0.0293 (17)	0.0031 (17)	0.0089 (14)	0.0020 (17)
C11	0.0324 (19)	0.018 (3)	0.0287 (17)	0.0041 (18)	0.0099 (15)	−0.0024 (17)
C12	0.044 (2)	0.022 (3)	0.037 (2)	0.005 (2)	0.0161 (17)	0.0047 (19)
C13	0.065 (3)	0.030 (3)	0.039 (2)	0.012 (2)	0.025 (2)	0.002 (2)
C14	0.066 (3)	0.030 (3)	0.033 (2)	0.002 (2)	0.012 (2)	−0.008 (2)
C15	0.048 (2)	0.027 (4)	0.041 (2)	−0.006 (2)	0.0107 (19)	−0.013 (2)
C16	0.037 (2)	0.033 (3)	0.042 (2)	0.001 (2)	0.0155 (18)	−0.008 (2)

### Geometric parameters (Å, º)

**Table:**

Re1—C1	1.905 (4)	C5—C6	1.374 (6)
Re1—C2	1.915 (4)	C5—H5	0.9400
Re1—C3	1.922 (6)	C6—C7	1.369 (6)
Re1—N2	2.164 (3)	C6—H6	0.9400
Re1—N1	2.201 (3)	C7—C8	1.380 (5)
Re1—Br1	2.6357 (5)	C7—H7	0.9400
O1—C1	1.153 (5)	C8—H8	0.9400
O2—C2	1.135 (4)	C10—C11	1.453 (5)
O3—C3	1.103 (6)	C11—C16	1.380 (6)
N1—C8	1.327 (5)	C11—C12	1.397 (5)
N1—C4	1.352 (5)	C12—C13	1.376 (6)
N2—C9	1.325 (4)	C12—H12	0.9400
N2—N3	1.361 (4)	C13—C14	1.373 (7)
N3—C10	1.353 (4)	C13—H13	0.9400
N3—H3	0.8700	C14—C15	1.371 (6)
N4—C10	1.328 (5)	C14—H14	0.9400
N4—C9	1.346 (5)	C15—C16	1.377 (6)
C4—C5	1.388 (5)	C15—H15	0.9400
C4—C9	1.442 (5)	C16—H16	0.9400
			
C1—Re1—C2	87.78 (17)	C7—C6—C5	119.4 (3)
C1—Re1—C3	90.4 (2)	C7—C6—H6	120.3
C2—Re1—C3	89.18 (19)	C5—C6—H6	120.3
C1—Re1—N2	168.91 (15)	C6—C7—C8	119.7 (4)
C2—Re1—N2	102.56 (14)	C6—C7—H7	120.2
C3—Re1—N2	93.71 (18)	C8—C7—H7	120.2
C1—Re1—N1	95.31 (14)	N1—C8—C7	122.0 (4)
C2—Re1—N1	176.88 (13)	N1—C8—H8	119.0
C3—Re1—N1	91.22 (15)	C7—C8—H8	119.0
N2—Re1—N1	74.33 (11)	N2—C9—N4	114.0 (3)
C1—Re1—Br1	91.81 (14)	N2—C9—C4	118.2 (3)
C2—Re1—Br1	93.80 (15)	N4—C9—C4	127.6 (3)
C3—Re1—Br1	176.38 (12)	N4—C10—N3	110.0 (3)
N2—Re1—Br1	83.63 (9)	N4—C10—C11	124.8 (3)
N1—Re1—Br1	85.69 (9)	N3—C10—C11	125.2 (3)
C8—N1—C4	118.4 (3)	C16—C11—C12	118.9 (4)
C8—N1—Re1	125.5 (3)	C16—C11—C10	122.9 (3)
C4—N1—Re1	116.1 (2)	C12—C11—C10	118.1 (3)
C9—N2—N3	103.7 (3)	C13—C12—C11	119.6 (4)
C9—N2—Re1	116.5 (3)	C13—C12—H12	120.2
N3—N2—Re1	139.3 (2)	C11—C12—H12	120.2
C10—N3—N2	108.5 (3)	C14—C13—C12	121.3 (4)
C10—N3—H3	125.7	C14—C13—H13	119.4
N2—N3—H3	125.7	C12—C13—H13	119.4
C10—N4—C9	103.8 (3)	C15—C14—C13	119.0 (4)
O1—C1—Re1	178.4 (4)	C15—C14—H14	120.5
O2—C2—Re1	175.6 (3)	C13—C14—H14	120.5
O3—C3—Re1	179.0 (4)	C14—C15—C16	120.8 (4)
N1—C4—C5	122.4 (4)	C14—C15—H15	119.6
N1—C4—C9	114.8 (3)	C16—C15—H15	119.6
C5—C4—C9	122.8 (3)	C15—C16—C11	120.4 (4)
C6—C5—C4	118.1 (4)	C15—C16—H16	119.8
C6—C5—H5	120.9	C11—C16—H16	119.8
C4—C5—H5	120.9		
			
C9—N2—N3—C10	−0.3 (4)	C5—C4—C9—N2	177.7 (4)
Re1—N2—N3—C10	170.7 (3)	N1—C4—C9—N4	172.3 (4)
C8—N1—C4—C5	1.1 (6)	C5—C4—C9—N4	−7.0 (7)
Re1—N1—C4—C5	−178.5 (3)	C9—N4—C10—N3	−0.7 (5)
C8—N1—C4—C9	−178.2 (4)	C9—N4—C10—C11	179.0 (4)
Re1—N1—C4—C9	2.2 (4)	N2—N3—C10—N4	0.7 (5)
N1—C4—C5—C6	0.0 (6)	N2—N3—C10—C11	−179.1 (4)
C9—C4—C5—C6	179.3 (4)	N4—C10—C11—C16	176.3 (4)
C4—C5—C6—C7	−1.0 (6)	N3—C10—C11—C16	−4.0 (7)
C5—C6—C7—C8	0.9 (7)	N4—C10—C11—C12	−2.2 (6)
C4—N1—C8—C7	−1.3 (6)	N3—C10—C11—C12	177.5 (4)
Re1—N1—C8—C7	178.3 (3)	C16—C11—C12—C13	−1.0 (7)
C6—C7—C8—N1	0.3 (7)	C10—C11—C12—C13	177.5 (4)
N3—N2—C9—N4	−0.1 (5)	C11—C12—C13—C14	0.0 (7)
Re1—N2—C9—N4	−173.6 (3)	C12—C13—C14—C15	0.8 (7)
N3—N2—C9—C4	175.8 (3)	C13—C14—C15—C16	−0.5 (7)
Re1—N2—C9—C4	2.3 (5)	C14—C15—C16—C11	−0.5 (7)
C10—N4—C9—N2	0.5 (5)	C12—C11—C16—C15	1.2 (7)
C10—N4—C9—C4	−174.9 (4)	C10—C11—C16—C15	−177.1 (4)
N1—C4—C9—N2	−3.0 (5)		

### Hydrogen-bond geometry (Å, º)

**Table:**

*D*—H···*A*	*D*—H	H···*A*	*D*···*A*	*D*—H···*A*
N3—H3···Br1^i^	0.87	2.51	3.360 (3)	168
C16—H16···Br1^i^	0.94	2.87	3.784 (4)	165
C6—H6···O2^ii^	0.94	2.38	3.194 (5)	145
C8—H8···O1^iii^	0.94	2.56	3.285 (5)	134

Symmetry codes: (i) −*x*+1/2, −*y*+1/2, −*z*; (ii) *x*+1/2, *y*+1/2, *z*; (iii) −*x*+1, *y*, −*z*+1/2.
